# Retraction: Metformin attenuates osteoclast-mediated abnormal subchondral bone remodeling and alleviates osteoarthritis via AMPK/NF-κB/ERK signaling pathway

**DOI:** 10.1371/journal.pone.0340042

**Published:** 2026-01-08

**Authors:** The PLOS One Editors

After this article [1] was published, concerns were raised about the results presented in Figs 3 and 7. Specifically, the following figure panels appear similar:

- Fig 3C RANKL 50 ng/mL + Met-0 μM day 3 panel of [1] and Fig 2A RANKL-50 ng/mL + DHA-5 μM panel of [2]; upon editorial follow-up, the second author stated that Fig 3C RANKL 50 ng/mL + Met-0 μM day 3 panel of [1] is incorrect.
- Fig 7A 4 weeks sham-operated panel* of [1] and Fig 7E left panel of [3, retracted in 4]; upon editorial follow-up, the second author noted they consider this does not affect the experimental conclusion because both panels report data for the sham-operated group in each study.
- Fig 7A 4 weeks DMM + Met panel* of [1] and Fig 7E right panel of [3].
- Fig 7A 8 weeks DMM + Met panel* of [1] and Fig 7E middle panel of [3], though it is noted some features appear to differ between the two panels.
- Fig 7B 8 weeks DMM + Met panel* of [1] and Fig 7A 8 weeks OA + CUR panel of [3].
- Fig 7C 4 weeks DMM + Met panel* of [1] and Fig 7B 4 weeks OA+vehicle panel of [3], though it is noted some features appear to differ between the differently stained tissue images.

PLOS additionally noted concerns about adherence to PLOS policy on Animal Research. Specifically, the ethical approval document submitted with [1] appears to reference a study title that does not align with the study described in the manuscript. Additionally, the approval document is dated after the study period during which the animal research was conducted, and the protocol period indicated in the ethics document is approximately one month, while the data presented in [1] span eight weeks. Upon editorial follow-up, the second author indicated that the study stemmed from a research project under a different title, and that the approved protocol period covered only the preparation and model creation stage at the Laboratory Animal Center, Ningxia Medical University, after which the intervention and sample collection work was carried out at the Key Laboratory of Fertility Preservation and Maintenance of Ministry of Education in Ningxia Medical University. During follow-up, raw data underlying the panels of concerns were provided; however, these data were not sufficient to resolve the concerns listed above. In light of the above unresolved concerns, the *PLOS One* Editors retract this article.

All authors agreed with the retraction and apologize for the issues with the published article.

Fig 3C RANKL 50 ng/mL + Met-0 μM day 3 panel appears similar to content in [2] under a CC BY 4.0 license by Spandidos Publications. Fig 3C RANKL 50 ng/mL + Met-0 μM day 3 panel is subject to the license that applied to the original article.
